# Pulmonary Delivery of Favipiravir in Rats Reaches High Local Concentrations without Causing Oxidative Lung Injury or Systemic Side Effects

**DOI:** 10.3390/pharmaceutics14112375

**Published:** 2022-11-04

**Authors:** Ozlem Akbal-Dagistan, Mustafa Sevim, Leyla Semiha Sen, Nur Sena Basarir, Meltem Culha, Aybige Erturk, Hanan Fael, Engin Kaptan, Serap Sancar, Lutfiye Mulazimoglu Durmusoglu, Berrak C. Yegen, Ayca Yildiz-Pekoz

**Affiliations:** 1 Department of Pharmaceutical Technology, Faculty of Pharmacy, Istanbul University, Istanbul 34116, Türkiye; 2 Department of Physiology, School of Medicine, Basic Medical Sciences, Marmara University, Istanbul 34854, Türkiye; 3 Department of Pharmaceutical Technology, Faculty of Pharmacy, Istinye University, Istanbul 34010, Türkiye; 4 Section of Molecular Biology, Department of Biology, Faculty of Science, Istanbul University, Istanbul 34116, Türkiye; 5 Department of Infectious Diseases, School of Medicine, Marmara University, Istanbul 34854, Türkiye

**Keywords:** favipiravir, antiviral, COVID-19, inhalation, pulmonary route, oxidative lung injury, hepatotoxicity, renal toxicity, cardiac toxicity

## Abstract

Favipiravir displays a rapid viral clearance, a high recovery rate and broad therapeutic safety; however, its oral administration was associated with systemic side effects in susceptible patients. Considering that the pulmonary route could provide a high drug concentration, and a safer application with less absorption into systemic circulation, it was aimed to elucidate whether favipiravir delivered via soft-mist inhaler has any deleterious effects on lung, liver and kidney tissues of healthy rats. Wistar albino rats of both sexes (n = 72) were placed in restrainers, and were given either saline or favipiravir (1, 2.5, 5 or 10 mg/kg in 1 mL saline) by inhalation within 2 min for 5 consecutive days. On the 6th day, electrocardiographic recording was obtained, and cardiac blood and lung tissues were collected. Favipiravir did not alter cardiac rhythm, blood cell counts, serum levels of alanine transaminase, aspartate transaminase, blood urea nitrogen, creatinine, urea or uric acid, and did not cause any significant changes in the pulmonary malondialdehyde, myeloperoxidase activity or antioxidant glutathione levels. Our data revealed that pulmonary use of favipiravir via soft-mist inhaler enables a high local concentration compared to plasma without oxidative lung injury or cardiac or hepatorenal dysfunction.

## 1. Introduction

The COVID-19 pandemic has necessitated the development of novel strategies to reduce and to prevent the high mortality and exaggerated transmission of the disease, as well as the overload of the healthcare systems [1]. During the pandemic, research has focused on the development of targeted vaccines and new antiviral drugs [2], while several antiviral drugs used in the treatment of various influenza sub-types and other RNA viruses, as well as other drugs commonly used with different indications, were extensively investigated for their efficiency against SARS-CoV-2 coronavirus infection [3,4]. Since discovering and developing new agents is a long-lasting and overwhelming process, the researchers have considered to reevaluate the existing drugs and their known methods of delivery for creating more facilitated solutions in the medical fight against the COVID-19 pandemic [5,6]. The antiviral drug favipiravir, which was shown to demonstrate a rapid viral clearance, a high recovery rate and broad therapeutic safety [7], is one of the leading drugs that has received approval for emergency use in treating SARS-CoV-2. Several studies have reported that favipiravir has a good safety profile with minor adverse reactions with no detectable effects on the QT/QTc interval [8,9,10,11,12,13,14]. However, there are reports demonstrating its detrimental effects on cardiac activity that lead to a prolonged QTc interval [15,16]. Moreover, case studies have suggested that favipiravir could trigger deterioration of hepatic and renal functions in susceptible patients [17,18,19]. Since clinical applications of favipiravir have so far involved its oral administration, it can be postulated that its local administration via the intrapulmonary route could reach the required treatment goals with less systemic side effects.

Administration of drugs via the pulmonary route provides a safer opportunity for the treatment of respiratory diseases, because possible drug–drug interactions and toxic effects on other organs are expected to be less due to reduced absorption of the drugs into the systemic circulation [6]. Moreover, when compared to other routes, a higher drug concentration can be locally reached by inhalation, consecutively providing a more rapid onset of the clinical response by overcoming the factors that would otherwise reduce the drug bioavailability (e.g., physiological barriers, poor gastrointestinal absorption and first-pass effect in the liver), while these therapeutic effects can be obtained by comparatively lower doses [20]. Since the SARS-CoV-2 coronavirus mainly affects the respiratory system, delivery of the drugs via the respiratory system was expected to enhance their therapeutic effects, while the possible systemic side effects could be eliminated. Accordingly, a newly developed antiviral drug triazavirin, which was delivered by aerosol inhalation to mice in a nose-only chamber, was shown to have a four-fold bioavailability than that was reached by its oral administration [21]. Similarly, remdesivir was formulated as dry powder for its possible use in COVID-19 patients by inhalation [22]. Using the small-particle aerosol form of a natural plant product (SP-303) was found to be effective in influenza A virus-infected mice and in rats with respiratory syncytial virus infection [23]. It is well known that the clinical effectiveness of an inhaled drug is closely related with the particle size and the distance at which it accumulates in the bronchial tree. Accordingly, soft-mist inhalers, which produce “aerosol clouds” with particle sizes ranging between 3–5 μm, enable more than 60% of the drugs to be delivered to the smaller bronchi and bronchioles [24]. Thus, based on the aforementioned studies highlighting that pulmonary administration of antiviral agents might provide novel treatment choices in the COVID-19 pandemic, the objective of the present study was to elucidate whether favipiravir delivered by soft-mist inhaler has any deleterious effects on the lung, liver and kidney tissues of healthy rats. If the use of this delivery method could be harmless but still effective, it would be then expected to have a potential for its utilization in treating most of the airborne viruses, including the SARS-CoV-2.

## 2. Materials and Methods

### 2.1. Animals and Chemicals

All experiments were conducted in compliance with the guidelines of the New York Academy of Sciences and the Turkish law on the use of animals in experiments. An ethical approval was obtained from the Marmara University Experimental Animals Ethics Committee (Date: 26 August 2020; No: 42.2020.mar). Rats (8–12 weeks old) were supplied by the İstanbul University Aziz Sancar Institute of Experimental Medicine. Experiments were initiated after a seven-day adaptation period following their transportation. The animals had free access to rat chow and water and were kept in standard conditions (22–24 °C) with 12-h cycles of light-dark.

A total of 72 Wistar albino male (300–350 g; n = 36) and female (160–250 g; n = 36) rats were randomly distributed in six groups consisting of six female and six male rats in each group. In order to determine the sample size, the resource equation method was used [25,26,27,28]. Since the small sample size may increase the possibility of false negatives and the study is an exploratory study, we used the 20/k + 1 (k: number of groups) formula (20/5 + 1 ~= 5). However, as some data were previously predicted to be non-parametric, the sample size was increased by approximately 1/3 [29]. Finally, due to the possibility of loss due to undesirable causes during the experiment, six animals per group were found to be sufficient for each gender by adding an extra animal to each group (6 × 2 × 6 = 72).

Favipiravir was provided at no charge by the Polifarma Drug Company (Polifarma & Aroma A.Ş. Istanbul, Turkey), while all the other chemicals were purchased from Sigma-Aldrich, St. Louis, MO, USA. Favipiravir stock solution was prepared at a concentration of 4 mg/mL in phosphate buffer saline (PBS) at pH 7.4 ± 0.05 (HANNA HI2002) and 300 ± 3 mOsm/kg (Osmometer, Model 2020, Advanced Instruments, Norwood, MA, USA) and 1.009 ± 0.1 mPa·s viscosity (Rheostress1, Thermo Scientific HAAKE MARS, Karlsruhe, Germany). Following the mechanical mixing process (IKA^®^Vortex Genius3, IKA-Werke GmbH, Staufen, Germany), the stock solution was then filtered through a membrane of 0.22 μm for sterilization.

### 2.2. Experimental Design

The rats were randomly distributed into five groups to receive either saline or favipiravir (1, 2.5, 5 or 10 mg/kg, diluted in 1 mL saline) by inhalation within 2 min. The chosen doses were determined based on the human-to-rat translation protocol [30]. First of all, considering that the amount of blood accumulated in the human lung is 10% at the resting conditions and 15% during exercise [31], the approximate amount that would reach the lungs was estimated. Secondly, the translation from human inhalation dose to rat inhalation dose was scaled as mentioned by Nair and Jacob [32]. Moreover, we recently reported that favipiravir exerts antiviral activity at the concentrations between 0.25 and 3 mg/mL on Vero-E6 that were infected with SARS-CoV-2/B.1.36 [33]. Then, the dosage was prepared in the appropriate volume to fill the dead space in the device and to reach approximately 50% accumulation in the lungs [34].

**Figure 1:**
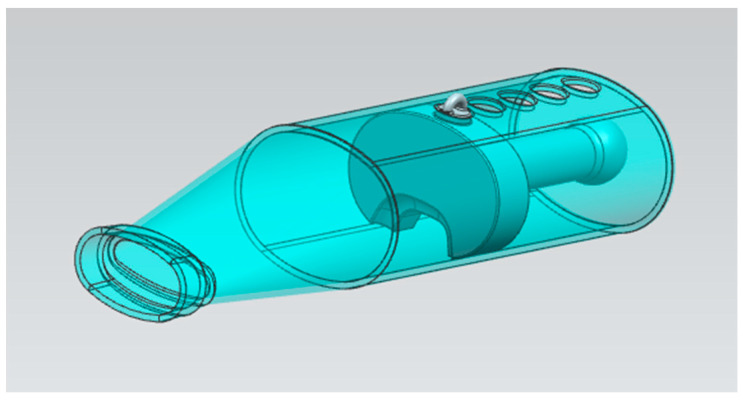
Design of the specially developed chamber adaptable to PulmoSpray^®^ for delivering treatment in rats.

Drug administration was conducted by soft-mist inhalers (PulmoSpray^®^ apparatus), which were kindly donated by Resyca BV (Enschede, The Netherlands), and were modified to be used in rats. When PulmoSpray^®^ is used in humans, conscious deep breathing allows the delivery of smaller aerosols to be effective. Since rats are not expected to take conscious deep breaths during the inhaler application, extra space and thereby extra distance for airflow was created to have the larger drug aerosols to be trapped, enabling the smaller aerosols to reach the lung. Using these modified PulmoSpray^®^ devices, favipiravir was delivered to the rats restrained in specially developed chambers (Figure 1). In order to reduce restraint stress and to enable effective inhalation, four different sizes of chambers for rats in different weights were designed and printed via a 3D printer.

The treatments were given twice a day for five consecutive days. On the 6th day (16 h after the last drug administration) cardiac blood was collected under anesthesia (ketamine, 100 mg/kg and xylazine hydrochloride, 10 mg/kg; intraperitoneally). In the rats treated with saline or with the highest dose (10 mg/kg) of favipiravir, electrocardiographic (ECG) recording of lead II (Carewell ECG-IIOIG Vet, Shenzhen, China) was performed under anesthesia before obtaining cardiac blood. RR and QT intervals were then measured to calculate the modified corrected and normalized Bazett formula (M-QTCn-B), which is based on RR intervals (average RR interval = 134.545 msn) [35]. Cardiac blood samples were used to determine the blood cell counts and serum levels of urea, uric acid, blood urea nitrogen (BUN), creatinine, aspartate transaminase (AST) and alanine transaminase (ALT) by spectrophotometric methods using an AU5800 automatic biochemical analyzer (Beckman Instruments, Fullerton, CA, USA). The lung tissues were excised and stored at −80 °C for the determination of myeloperoxidase (MPO) activity, malondialdehyde (MDA) and glutathione (GSH) levels. Extra plasma and lung tissue samples of the rats treated with the highest dose (10 mg/kg) of favipiravir were stored at −80 °C for the determination of the drug levels by high liquid pressured chromatography (HPLC). Additional lung samples were stored in paraformaldehyde for histological analyses.

### 2.3. Measurement of Myeloperoxidase Activity in Lung Tissue Samples

Since myeloperoxidase (MPO) is an enzyme that is found primarily in the azurophilic granules of polymorphonuclear leukocytes (PMNL), MPO activity measurement is commonly used to estimate tissue PMNL accumulation in inflamed tissues [36]. For this purpose, after washing tissues with phosphate-buffered saline (PBS), lung tissue samples were homogenized by detergent buffer (in 50 mM potassium phosphate, 10 mM EDTA, 0.5% HETAB at pH 6.0), then centrifuged at 4 °C and 12,000 rpm for 10 min. After the supernatant was removed, the pellet was homogenized again in a 50 mM PBS. MPO activity was determined by spectrophotometric measurement of the 2 HCl (20 mg/mL)—dependent oxidation of o-dianizidine to H_2_O_2_- (20 mM), measured at 460 nm wavelength at 37 °C, and the MPO activity was expressed as U/g.

### 2.4. Measurement of Lipid Peroxidation and Glutathione Levels in Lung Tissue Samples

Malondialdehyde (MDA) is one of the secondary products of lipid peroxidation used as an indicator of cell membrane injury, while glutathione (GSH) is a cellular antioxidant that combats against reactive oxygen metabolites. Tissue samples were homogenized in a 10% solution of trichloracetic acid and were centrifuged at 3000 rpm, 4 °C for 15 min. After the centrifugation, 750 µL of the supernatant was used for determining lipid peroxidation, and 250 µL was used for GSH measurement. Briefly, after centrifugation, 2-thiobarbituric acid was added to the supernatant (750 µL) and put in a boiling water bath at 90 °C for 30 min. In measuring GSH, the modified Ellman method was used. Briefly, 1 mL of 0.3 mol/L Na_2_HPO_4_·H_2_O was added to 250 µL of supernatant. Then, 125µL of 5,5′-dithiobis (2-nitrobenzoic acid) (0.4 mg/mL in 1% sodium citrate solution) was added to this mixture. The amount of MDA was read using a spectrophotometer at 535 nm absorbance and the results were expressed in nmol/g/tissue, while the amount of GSH was read at 412 nm absorbance and the results of GSH were expressed in µmol/g/tissue [37,38,39].

### 2.5. Measurement of Favipiravir Level in the Plasma and Lung Tissue Samples

In another group of rats (n = 12, 6 female and 6 male) favipiravir was delivered at the highest dose (10 mg/kg), and the lungs of animals were immediately removed for the determination of the pulmonary amount of favipiravir. The lung was minced into small pieces, parts were accurately weighed, and transferred into tubes that contains 0.5 mL PBS. Samples were homogenized using MagNA Lyser Instrument at 7000 rpm for 30 s for three cycles with MagNA Lyser Green beads (Roche Diagnostics GmbH, Mannheim, Germany). The homogenized tissue was precisely transferred into a 2 mL Eppendorf tube and methanol was added into each tube and vortexed for 1 min and then centrifugated at 15,000 rpm and 4 °C for 10 min. Aliquots of the supernatant was diluted again with methanol and the vortex and centrifugation steps were repeated. The resulted supernatant was collected and proceeded for HPLC analysis [32].

HPLC analysis was performed on a SHIMADZU system equipped with photodiode array detector, autosampler, quaternary pump and column oven. The analysis was achieved by applying a previously published method applied for quantification of favipiravir in spiked plasma using C18 column (250 mm × 4.6 mm, 5 μm) at 30 °C. The mobile phase consisted of 10 mM sodium phosphate buffer pH 6.9 (solution A) and methanol (solution B). Separation was achieved using gradient elution at a flow rate of 1.0 mL min-1. The injection volume was 10 μL and the detection wavelength was 364 nm. This method was validated for linearity, specificity, precision and accuracy over the concentration range of 0.5–50 μg mL^−1^ [40].

### 2.6. Histological Analysis

The lung tissue samples were fixed in Bouin’s fixative solution for 24 h. Tissues were washed with 70% ethanol to remove fixative, then kept in ascending ethanol series (90%, 96%, 2 × 100%) to remove water from the tissues and were embedded in paraffin after clearing in xylene. Serial sections at 5-µm thickness were taken using a rotary microtome (Leica, Germany). Sections adhered to the slide were then dried in an oven at 37 °C for at least 24 h. The sections dried on slides were stained with hematoxylin-eosin (H&E) and examined under a light microscope (Olympus, Cx23, Tokyo, Japan). Histological damage score was calculated by scoring pulmonary edema, alveolar damage, inflammatory cells in bronchial lumens, alveolar inflammatory cell infiltration, interstitial fibrosis, lymphoid vascular invasion, pulmonary congestion, thrombosis and hemorrhage. The degree of histological damage was scored using a scale of 0 to 3 (0: absent, 1: mild, 2: moderate, 3: severe), where the maximum score that could be given was 27. The photomicrographs of stained lung sections were taken at ×200 magnification using a photomicroscope (Olympus, Bx53, Tokyo, Japan).

### 2.7. Statistical Analysis

All the statistical analyses were performed using GraphPad Prism 9.2.0 (GraphPad Software, San Diego, CA, USA). The normality of the distribution of the data was tested using Shapiro–Wilk, and parametric tests were used for data that were distributed normally. The data were expressed as mean ± standard deviation. Comparisons among different groups were analyzed by unpaired *t*-test or one-way ANOVA followed by post-hoc Dunnett multiple comparison tests. Values of *p* < 0.05 were regarded as significant.

## 3. Results

### 3.1. Effect of Inhaled Favipiravir on Blood Cell Counts and Hepatic and Renal Function Tests

**Figure 2:**
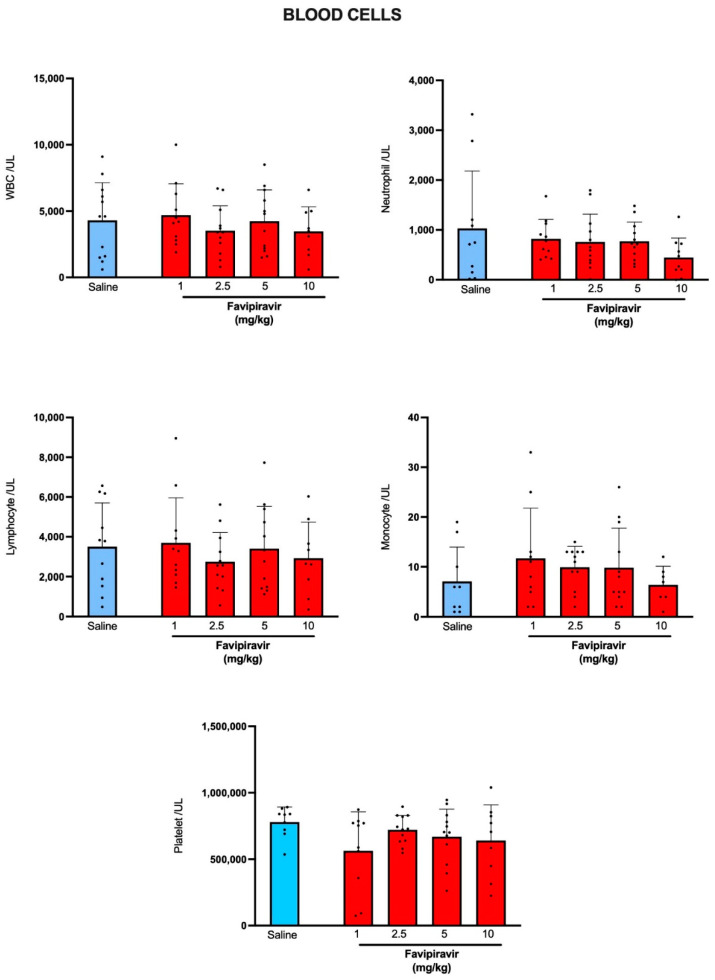
Blood cell counts in rats treated twice a day for five consecutive days with saline or four doses (1, 2.5, 5 and 10 mg/kg) of favipiravir via the soft-mist inhalers. Data are expressed as mean ± standard deviation (SD); n = 12 in each group. Ordinary one-way ANOVA followed by post-hoc Tukey test was used. No statistical difference was observed between the groups.

**Figure 3:**
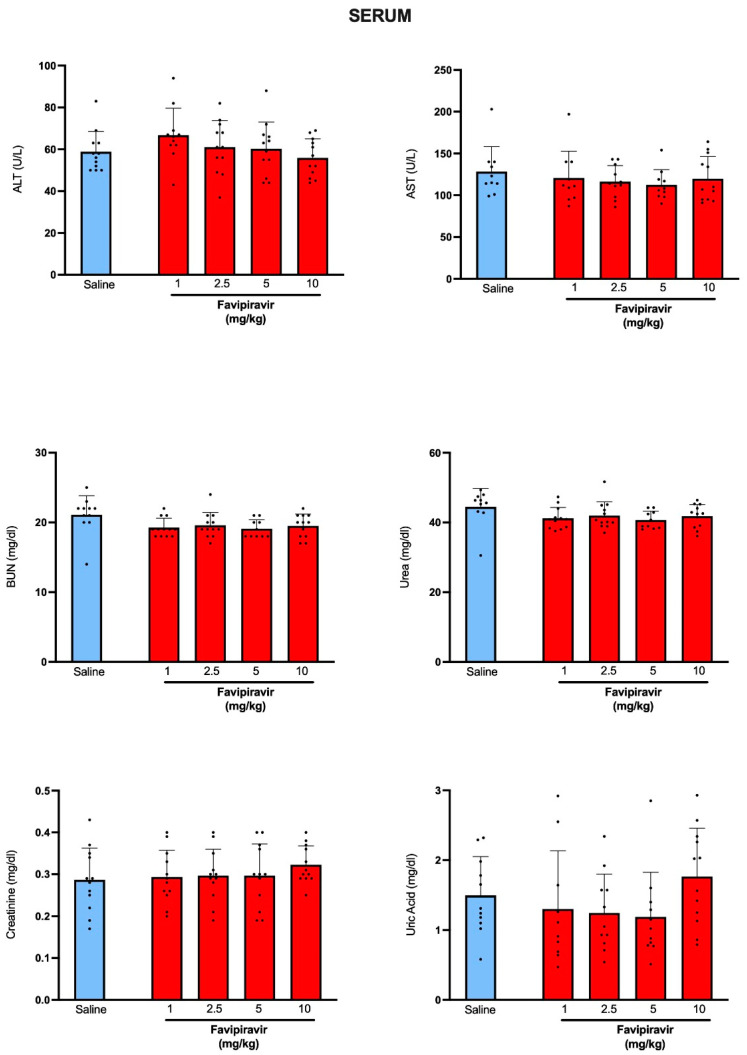
Serum levels of alanine transaminase (ALT), aspartate transaminase (AST), blood urea nitrogen (BUN), urea, creatinine and uric acid in rats treated twice a day for five consecutive days with saline or four doses (1, 2.5, 5 and 10 mg/kg) of favipiravir via the soft-mist inhalers. Data are expressed as mean ± standard deviation (SD); n = 12 in each group. Ordinary one-way ANOVA followed by post-hoc Tukey test was used. No statistical difference was observed between the groups.

When compared with the blood cell counts of rats that have received saline by soft-mist inhalers, favipiravir application by soft-mist inhaler at none of its used doses has altered total white blood cell count or the counts of neutrophils, lymphocytes, monocytes or platelets (Figure 2). As indicators of normal hepatic function, serum ALT and AST levels of favipiravir-treated rats were not different than those of the saline-treated group (Figure 3). BUN and serum levels of creatinine, urea and uric acid levels were also similar in saline-treated and favipiravir-treated groups, showing that favipiravir application by soft-mist inhaler has not altered renal function tests.

### 3.2. Effect of Inhaled Favipiravir on Levels of Malondialdehyde, Glutathione, Myeloperoxidase Activity and Histological Appearance of Lung Tissues

**Figure 4:**
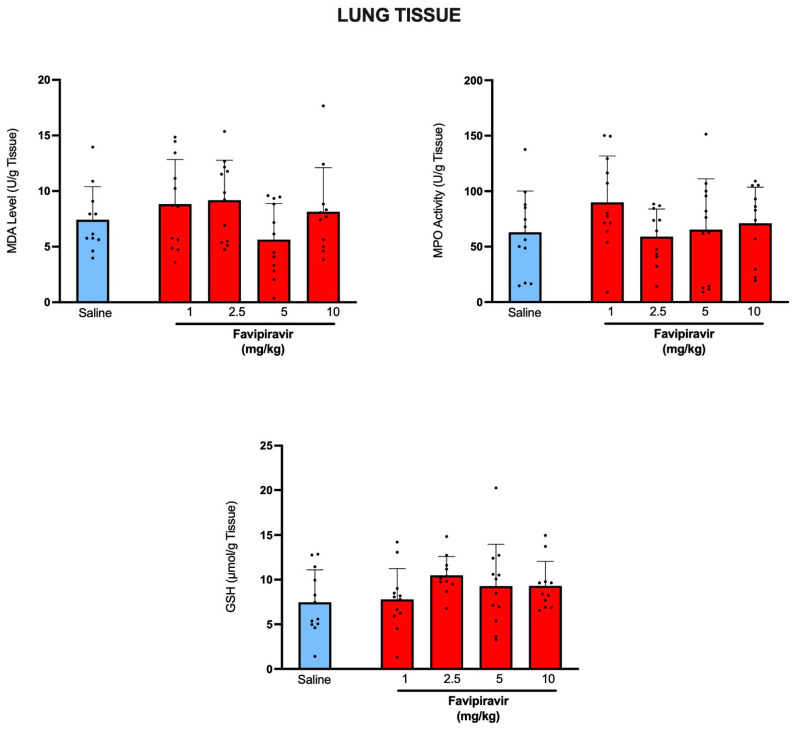
Levels of lipid peroxidation (MDA), myeloperoxidase (MPO) activity and antioxidant glutathione (GSH) levels in the lung tissues of rats treated twice a day for five consecutive days with saline or four doses (1, 2.5, 5 and 10 mg/kg) of favipiravir via the soft-mist inhalers. Data are expressed as mean ± standard deviation (SD); n = 12 in each group. Ordinary one-way ANOVA followed by post-hoc Tukey test was used. No statistical difference was observed between the groups.

Delivery of favipiravir by soft-mist inhaler, when compared with saline delivery has not resulted in significant changes in lipid peroxidation (MDA) or antioxidant GSH levels, while MPO activity levels were also similar in all favipiravir-treated and saline-treated groups (Figure 4), showing that local administration of favipiravir has not resulted in oxidative injury of lung tissue.

**Figure 5:**
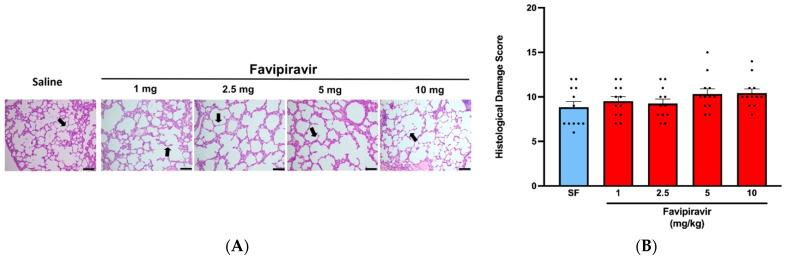
Histological appearances (**A**) and damage scores (**B**) in the lung tissues from rats treated twice a day for five consecutive days with saline or four doses (1, 2.5, 5 and 10 mg/kg) of favipiravir via the soft-mist inhalers. The scale bar indicates 200 μm for the control image, 100 μm for the experimental images. Data are expressed as mean ± standard deviation (SD); n = 12 in each group. Ordinary one-way ANOVA followed by post-hoc Tukey test was used. No statistical difference was observed between the groups.

Light microscopic examination of both saline-treated and favipiravir-treated groups exhibited normal lung histology with thin alveolar walls and varying sizes of alveolar sacs and well-organized alveolar channels (Figure 5A), and the histological damage scores were not significantly different among experimental groups (Figure 5B).

### 3.3. Plasma and Lung Tissue Levels of Favipiravir and QT Intervals on ECG

**Figure 6:**
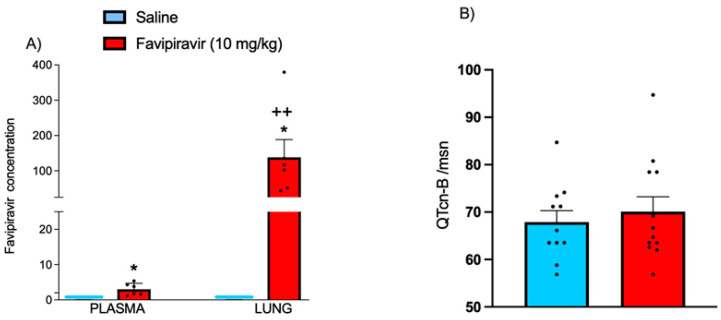
Favipiravir levels determined by HPLC analysis in the plasma (3.0 ± 0.71 µg/mL) and lung tissues (138.2 ± 50.5 µg/g) (n = 6) (**A**), and the calculated cardiac QTCn-B values (n_saline_ = 11, n_favipiravir_ = 12) (**B**) of rats treated twice a day for five consecutive days with saline or four doses (1, 2.5, 5 and 10 mg/kg) of favipiravir via the soft-mist inhalers. Data are expressed as mean ± standard deviation (SD). Ordinary one-way ANOVA followed by post-hoc Tukey test and Student’s *t*-test were used. * *p* < 0.05, compared to saline-treated group; ++ *p* < 0.01, compared to plasma level of favipiravir-treated group.

In plasma and lung tissue samples, which were obtained immediately after the last administration of 10 successive favipiravir (10 mg/kg) delivery sessions by soft-mist inhaler, favipiravir was detected at significantly elevated levels (Figure 6A). The concentration measured in the lung tissue was 140 µg/g lung tissue which was more than 45-folds compared to plasma, showing that the local administration of favipiravir has resulted in a higher accumulation in the lung tissue. At the 10 mg/kg dose of favipiravir, calculated QT intervals were not significantly different than those of the saline-treated rats, showing that the highest dose has not resulted in the prolongation of QT interval (Figure 6B).

## 4. Discussion

Our results demonstrated that favipiravir, when administered using soft-mist inhaler, did not alter the cardiac rhythm, blood cell counts, serum levels of ALT, AST, BUN, creatinine, urea or uric acid, and did not result in significant changes in the pulmonary malondialdehyde, myeloperoxidase activity or antioxidant glutathione levels. Furthermore, our data revealed that the local administration of favipiravir using soft-mist inhaler enabled a higher local concentration of the drug without causing oxidative lung injury or cardiac or hepatorenal dysfunction.

Since favipiravir was known to exert antiviral activity against many RNA viruses [41], it was considered as a promising drug for SARS-CoV-2. Although in vitro studies have suggested that favipiravir would be a suitable candidate for the treatment of COVID-19 [42], it was shown that comparatively higher concentrations of favipiravir were required to reduce the viral infection in Vero E6 cells [43]. In hamsters infected with a variety of viruses, an orally given low-dose (300 mg·kg^−1^·d^−1^) of favipiravir was found to be effective, but the same dose was not efficient in SARS-CoV-2-infected hamsters [44,45,46]. When the medium and high doses of favipiravir were used, histologically significant improvements in lung pathology were reported, which revealed that peribronchial and perivascular inflammation and bronchopneumonia were milder at the medium dose and nearly absent at the highest dose [47]. Likewise, another study on the analysis of pulmonary histopathological changes revealed that favipiravir played a protective role by reducing the severity of the lesions [48]. Several clinical trials have also been initiated to evaluate the efficacy and safety of favipiravir in COVID-19 patients [12]. Earlier clinical studies have shown that favipiravir has therapeutic effects on COVID-19 patients in terms of disease progression and viral clearance [49] and has shortened the latency to relief for fever and cough [50]. Therefore, treatment guidelines of many countries have included favipiravir in the treatment protocol of COVID-19 disease, and its oral tablet form has been commonly used In COVID-19 patients. However, recent clinical studies on the efficacy of favipiravir in COVID-19 have shown that favipiravir does not provide a significant beneficial effect [51,52]. Considering these conflicting data, we have hypothesized that the orally administered favipiravir was not able to reach the therapeutic level in the lungs to inhibit SARS-CoV-2 replication. In support of our prediction, it was demonstrated that the radiolabeled favipiravir, upon its oral or intravenous administration, was least distributed in the lungs of mice [53]. Accordingly, low pulmonary distribution of orally administered lopinavir–ritonavir in rats [54] was suggested to be responsible for insufficient concentration in the lungs to inhibit SARS-CoV-2 replication and the cause of the failure of lopinavir treatment in COVID-19 patients [55]. Similarly, it was recently stated that an intravenous dose of remdesivir was not able to achieve the sufficient concentrations to kill SARS-CoV-2 in the human lung and its direct pulmonary administration could provide higher drug concentration in the lung and reduce systemic toxicity in COVID-19 patients [56]. Thus, in parallel with these reports, our recent findings verified that the pulmonary administration of favipiravir yields to higher concentration of the drug in the lung tissue as compared to plasma, which may be critical for the improved recovery of COVID-19 patients. Delivery of the drugs via the respiratory route is expected to enhance therapeutic effects of the drugs by providing high drug accumulation with comparatively lower doses, which also helps to avoid systemic toxicity. Initially, nebulizers were regarded as the most suitable devices for the continuous delivery of medication in COVID-19 patients [57,58], but the spread of respirable droplets by nebulizers was shown to increase viral transmission and, in turn, to risk healthcare providers [59,60]. Traditional inhaler devices, such as metered-dose inhalers (MDIs) and dry powder inhalers (DPIs) have some limitations and a more convenient propellant-free inhaler is required to allow effective delivery of aerosols from solutions. These requirements have prompted the development of soft mist technology, which is able to generate a single-breath, inhalable aerosol from a drug solution [61]. Soft-mist inhalers are more desirable for drug targeting to the lungs compared to MDIs and DPIs, and the generation of drug aerosol is independent of the inspiratory capacity of the patient [62]. Moreover, soft-mist inhalers provide superiority with their easy use, patient-based dose setting and closed mouthpiece technology that prevents the spread out of contaminated aerosol droplets [60,63,64], making it advantageous for their use in the pulmonary infectious diseases. In our previous study performed to evaluate the in vitro antiviral activity of favipiravir, it was concluded that favipiravir at a dosage of 2 mg/mL effectively inhibits the SARS-CoV-2 virus [33]. The follow-up preclinical data support the claim of the study that the inhalation route provides higher lung accumulation compared to plasma. Additionally, another study that was conducted by El Azab et al. on rats showed that after intraperitoneal application of favipiravir (8 mg/kg) the Cmax value was calculated as 31.5 µg [65]. Likewise, studies that were performed on favipiravir have shown low lung intake concentrations [53]. On the other hand, the study performed in this manuscript has chosen a different drug delivery approach that has allowed the uptake value to reach 140 µg/mg in the lungs following inhalation administration. Given the high doses (1600 mg–3600 mg) that were recommended orally by the treatment guidelines, the fact that a modest dose, like 2 mg/mL, may achieve antiviral activity in the lungs could be a sign of the superiority of an inhalation formulation.

Although it was considered that the adverse effects caused by favipiravir are mild and manageable [50], elevated levels of uric acid, ALT, AST and triglyceride and decreased neutrophil count were evident in favipiravir-treated patients [66]. Favipiravir-associated significant bradycardia with prolongation of QT interval was reported in COVID-19 patients [67,68]. It has been reported that hyperuricemia is the most frequent side effect of favipiravir [69], which requires cautiousness when favipiravir is to be used for patients with elevated blood uric acid, history of gout or kidney dysfunction [70]. Clinically, disrupted liver function test indicative of liver damage was considered as a frequently observed critical side effect of favipiravir [71,72]. Similarly, serum levels of AST, ALT, urea, creatinine and C-reactive protein (CRP) concentrations were elevated and hepatorenal injury was documented histologically in rats when favipiravir at 200 mg/kg dose was orally administered for five days [73]. It was also shown that oral administration of favipiravir in rats at a dose of 200 mg/kg for 10 days suppressed the erythrocytes, lymphocytes and monocytes [74]. On the other hand, our findings showed that pulmonary application of favipiravir at 10 mg/kg twice a day for five days tended to increase the blood uric acid level, but no statistical significance was observed, while neither the blood cell counts nor the hepatic and renal function tests were altered by pulmonary applications of favipiravir (1–10 mg/kg). Since increased levels of liver enzymes have been directly associated with COVID-19 [75] and we have applied favipiravir to healthy uninfected animals, it is possible to postulate that the hepatic side effects of favipiravir may be due to complexity of the COVID-19 infection itself. Furthermore, no change in QT interval was evident by the five-day favipiravir treatment using a soft-mist inhaler. These results suggest that choosing the optimum dose of favipiravir and its local application may be critical in the prevention of its systemic side effects.

Ever since the COVID-19 pandemic has established the obligatory usage of masks, many respiratory tract viruses did not have the appropriate environment to get spread, which should have caused an immunity attenuation for common respiratory viruses. Consequently, the world should be ready for the fight against epidemics with those viruses. Despite conflicting data of the efficacy of favipiravir for the treatment of SARS-CoV-2 infection, it is very obvious that viruses and antiviral treatment would be one of the most important issues in the infectious diseases area [76,77]. The anti-influenza activity of favipiravir is well known and has been confirmed in cell culture experiments and animal models as well as clinical trials. Moreover, favipiravir is also effective against other RNA respiratory viruses and may be a candidate for the treatment of serious infections caused by human rhinovirus, respiratory syncytial virus, metapneumovirus, parainfluenza viruses and hantavirus pulmonary syndrome [78]. Thus, the safe pulmonary delivery of favipiravir could be a strong candidate for the treatment of not only SARS-CoV-2, but also against other RNA respiratory viruses awaiting in the future.

## 5. Conclusions

Our data revealed that the pulmonary use of favipiravir using a soft-mist inhaler enables a higher local concentration with respect to plasma without causing oxidative lung injury or cardiac or hepatorenal dysfunction. Clinical trials are required to assess the therapeutic potential of favipiravir in the lungs for both SARS-CoV-2, and additional RNA respiratory viruses. As a result, in the new era of “antimicrobial drug should be delivered to the site of infection”, the soft-mist inhaler promises a good drug delivery option for favipiravir and a possible economical approach “less drug, less adverse effects, less drug-drug interactions” for the potential treatment of many respiratory viruses.

## Abbreviations

EC_50_	half-maximal effective concentration
CC_50_	half-cytotoxic concentration
SI	selectivity index
b.i.d	twice a day
q.d.	once a day
